# Advances in pathophysiological mechanisms and therapeutic efficacy of exercise rehabilitation in patients with heart failure with preserved ejection fraction

**DOI:** 10.3389/fcvm.2025.1598878

**Published:** 2025-05-27

**Authors:** Juanjuan Fang, Zhenhua Wang, Jiangshui Yu

**Affiliations:** The Second Affiliated Hospital of Fujian Medical University, Quanzhou, Fujian, China

**Keywords:** heart failure with preserved ejection fraction, pathophysiological mechanisms, exercise rehabilitation, exercise intolerance, therapeutic evidence, optimization strategies

## Abstract

Heart Failure with Preserved Ejection Fraction (HFpEF) is a heterogeneous syndrome characterized by systemic multiorgan dysfunction, and exercise rehabilitation has emerged as a promising non-pharmacological intervention. This review synthesizes current evidence on the pathophysiological mechanisms underlying exercise intolerance in HFpEF and evaluates the therapeutic efficacy of exercise-based interventions. Key mechanisms include myocardial stiffness due to chronic inflammation, coronary microvascular dysfunction, skeletal muscle mitochondrial impairment, and endothelial dysfunction. Clinical studies indicate that tailored exercise regimens (e.g., combined aerobic-resistance training) improve peak oxygen consumption, 6 min walking distance, and quality of life through multi-organ adaptations: enhanced cardiac output reserve, skeletal muscle metabolic remodeling, and reduced systemic inflammation. However, challenges persist in optimizing exercise prescriptions for phenotypically diverse HFpEF subpopulations (e.g., obese, elderly frail). Future research must prioritize phenotype-specific protocols, validate long-term outcomes (mortality, hospitalization), and integrate biomarkers (e.g., H_2_FPEF score) with digital health technologies to advance precision rehabilitation strategies. This review highlights the imperative for mechanistic insights to guide clinical translation in HFpEF management.

## 1 Introduction

Heart failure with preserved ejection fraction (HFpEF), is defined as a left ventricular ejection fraction (LVEF) ≥50% with accompanying symptoms and/or signs, in the presence of objective evidence of cardiac structural and/or functional abnormalities consistent with the presence of LV diastolic dysfunction/raised LV filling pressures, including raised natriuretic peptides (1). HFpEF constituting nearly 50% of heart failure cases, is a multisystem disorder driven by aging, obesity, and metabolic dysfunction (1–3). Unlike heart failure with reduced ejection fraction (HFrEF), HFpEF involves systemic pathophysiology such as myocardial stiffness, skeletal muscle mitochondrial impairment, endothelial dysfunction, and neurohormonal activation, culminating in profound exercise intolerance and poor prognosis (4–6). Despite pharmacological advances, no therapies improve survival, underscoring the unmet need for effective interventions (7). Exercise rehabilitation emerges as a pivotal non-pharmacological strategy, demonstrating improvements in functional capacity of peak oxygen consumption (VO_2_peak), quality of life, and hemodynamic profiles through cardiac-skeletal muscle adaptations and anti-inflammatory effects (8, 9). However, evidence gaps persist: most trials focus on short-term outcomes (3–6 months), while impacts on mortality/hospitalization remain unproven (10, 11). HFpEF's heterogeneity, obese, elderly, or amyloidosis subphenotypes, demands precision approaches to optimize efficacy-safety balances (12, 13). This review synthesizes mechanisms of exercise intolerance, evaluates therapeutic evidence, and proposes a roadmap integrating phenomapping, digital monitoring, and tailored regimens to transform HFpEF rehabilitation from symptom management to disease modification.

## 2 Epidemiological characteristics of HFpEF

HFpEF constitutes approximately 50% of heart failure cases, with rising prevalence linked to aging, obesity, and metabolic comorbidities (2, 3). Large cohort studies show comparable HFpEF/HFrEF incidence (4). Women exhibit higher HFpEF risk, tied to estrogen signaling and pregnancy complications (e.g., preeclampsia) (5, 6).

Independent risk factors for HFpEF include advanced age, obesity, diabetes, hypertension, and atrial fibrillation(AF) (1). Current smoking shows dose-response HFpEF/HFrEF risk. Cessation reduces but residual risk persists decades post-cessation (7). Infertility history, also elevate HFpEF risk (8). Racial disparities are evident, with African American populations showing heightened left ventricular hypertrophy and concentric remodeling, predisposing them to HFpEF (9).

HFpEF manifests as a multisystem disorder, involving skeletal muscle dysfunction, peripheral vascular abnormalities, pulmonary congestion, renal impairment, and cerebral hemodynamic alterations (1). Comorbid cardiovascular conditions, including secondary tricuspid regurgitation (STR) and pulmonary hypertension (PH), are prevalent. Approximately 35% of severe STR cases are attributable to HFpEF, with concomitant STR increasing adverse event risks (10). HFpEF also correlates strongly with stroke; post-stroke patients exhibit elevated HFpEF hospitalization rates and cardiovascular event incidence (11).

## 3 Pathophysiological characteristics of HFpEF and mechanisms of exercise intolerance

### 3.1 Pathophysiological characteristics of HFpEF

**Figure 1:**
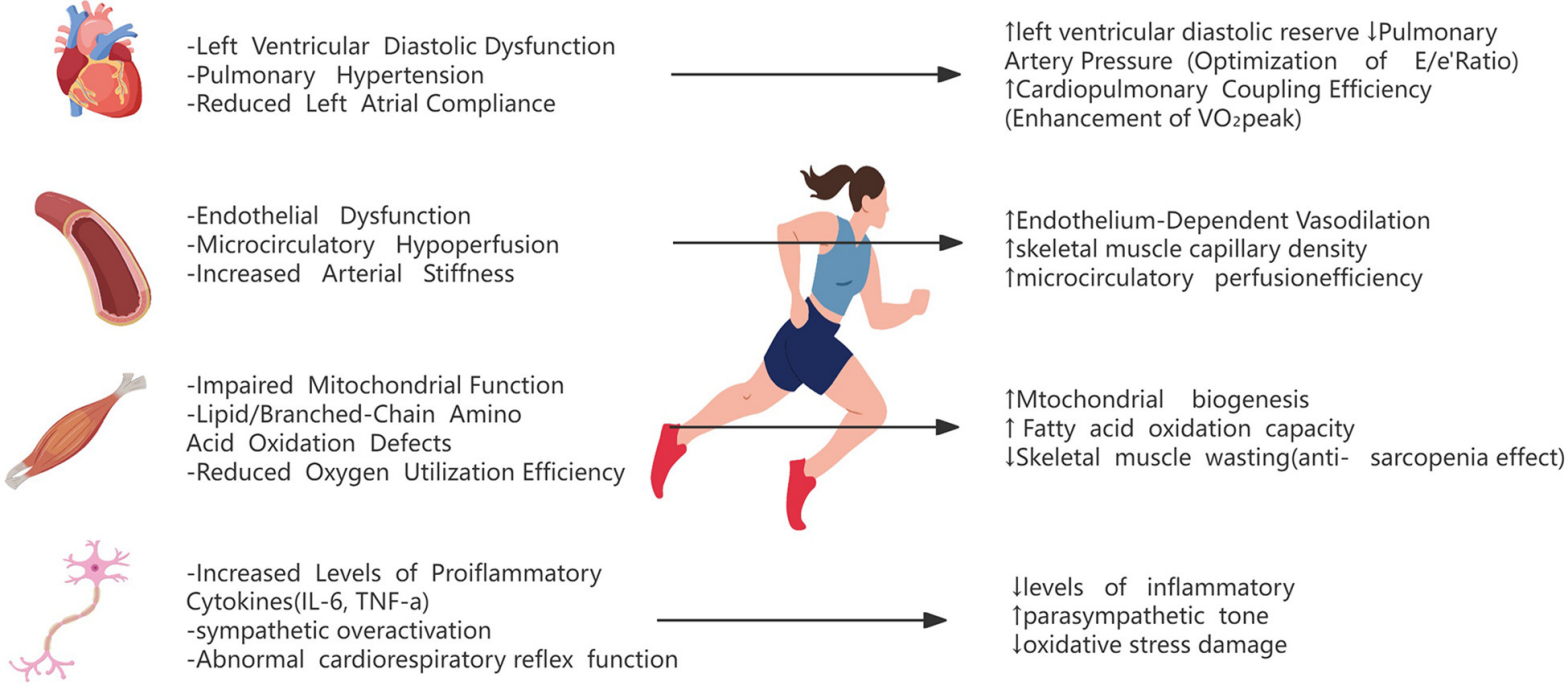
Pathophysiological mechanisms of exercise intolerance and exercise interventions in HFpEF.

The pathophysiological landscape of HFpEF is characterized by multisystem organ involvement, extending beyond cardiac dysfunction to encompass skeletal muscle metabolic derangements, pulmonary vascular congestion, renal impairment, peripheral endothelial dysfunction, and neurovascular dysregulation (2) (Figure 1). Central to its pathogenesis is the chronic low-grade inflammation and metabolic dysregulation. Obesity, diabetes, and hypertension drive visceral adipose tissue (VAT) and epicardial adipose tissue (EAT) expansion, which secretes proinflammatory cytokines [e.g., Interleukin-6 (IL-6), Tumor Necrosis Factor-alpha (TNF-α)] and profibrotic mediators, ultimately inducing myocardial stiffness augmentation and diastolic impairment (12).

Coronary microvascular dysfunction (CMD) affects 50% of HFpEF patients, driving myocardial ischemia, calcium mishandling, and impaired relaxation (13). A multicenter prospective cohort study demonstrated that 91% of HFpEF patients exhibited either epicardial coronary artery disease (CAD), CMD, or both. Among those without obstructive CAD, >80% displayed endothelium-independent or endothelium-dependent CMD (13).

Structural cardiac remodeling, including left ventricular hypertrophy (LVH) and left atrial myopathy, further typifies HFpEF. The hemodynamic hallmark of HFpEF, elevated left ventricular filling pressures and exertional intolerance, primarily stems from left ventricular diastolic dysfunction, arising from impaired relaxation kinetics due to dysregulated sarcoplasmic reticulum calcium reuptake (SERCA2a dysfunction) (14), cardiomyocyte hypertrophy with altered titin isoform expression, and extracellular matrix (ECM) remodeling via collagen crosslinking (15, 16), compounded by left atrial (LA) decompensation manifested as reduced LA compliance from interstitial fibrosis (17), impaired LA reservoir/conduit function (18), and diminished left atrial emptying fraction with compliance reduction—all correlating with elevated pulmonary capillary wedge pressure(PCWP) (19).

Notably, transthyretin amyloid cardiomyopathy (ATTR-CM) demonstrates high prevalence among elderly HFpEF cohorts, with cardiac amyloid deposition directly compromising diastolic mechanics through myocyte infiltration and restrictive physiology (19).

### 3.2 Mechanisms of exercise intolerance in HFpEF

The mechanistic basis of exercise intolerance in HFpEF arises from multilevel pathophysiological derangements, with skeletal muscle dysfunction constituting an essential component. Impaired skeletal muscle bioenergetics—characterized by reduced oxidative capacity, diminished mitochondrial content, and aberrant mitochondrial dynamics (fusion/fission imbalance)—significantly contributes to exertional limitation in elderly HFpEF cohorts (20). Skeletal muscle phenotype switching further exacerbates functional decline, evidenced by selective reduction of type I oxidative muscle fibers (reliant on mitochondrial ATP production) in HFpEF patients (21). Mitochondrial dysfunction manifests as network fragmentation, decreased mitochondrial cross-sectional area, and downregulation of fusion regulators (Mitofusin 1, Mitofusin 2, Optic atrophy 1), collectively impairing oxidative phosphorylation capacity (22, 23). These defects potentiate calcium mishandling, oxidative stress overload, and nitric oxide (NO) depletion, driving endothelial/cardiomyocyte uncoupling (24). Concomitant obesity-related myosteatosis and muscle atrophy further compromise oxygen utilization efficiency (25, 26).

Abnormal cardiopulmonary interactions exacerbate hemodynamic compromise through three interlinked mechanisms: (1) Diastolic reserve exhaustion during exertion elevates left atrial pressure, precipitating post-capillary PH via pulmonary venous congestion—a phenotype observed in approximately 80% of HFpEF patients (27, 28); (2) This PH-driven right ventricular afterload augmentation disrupts ventilation-perfusion (V/Q) matching through altered pulmonary vascular impedance and right-left ventricular interdependence (2, 19); (3) Concomitant inspiratory muscle weakness, independent of cardiac loading conditions, directly correlates with reduced exercise capacity by impairing respiratory pump efficiency and oxygen delivery (29).

Peripheral vascular dysfunction encompasses two interrelated pathological axes: firstly, endothelial-dependent vasodilatory impairment driven by reduced nitric oxide (NO) bioavailability restricts microvascular reserve capacity during exertion (30); concurrently, arterial stiffening—quantified invasively through elevated aortic impedance—exacerbates ventricular-arterial uncoupling, thereby diminishing peak oxygen consumption (VO2peak) (7, 31). Critically, exercise-induced exacerbation of arterial stiffness demonstrates a direct linear association with pathological increments in pulmonary capillary wedge pressure (PCWP) during exertion, thereby contributing to diminished peak oxygen uptake (VO2peak) through ventricular-arterial decoupling and impaired cardiopulmonary efficiency (32).

Autonomic dysregulation perpetuates this vicious cycle through three sequential pathological cascades: Initially, sympathetic nervous system and renin-angiotensin-aldosterone system overactivation initiates a maladaptive cascade—inducing vasoconstrictive responses and aldosterone-mediated myocardial fibrosis (33); subsequently, chronic norepinephrine excess triggers β-adrenergic receptor downregulation via GRK2-mediated desensitization, while concurrently promoting cardiomyocyte apoptosis through calcium/calpain pathway activation (34); compounding these effects, hypoxia-induced lipotoxic metabolites (e.g., free fatty acids) directly inhibit mitochondrial complex I/III activity, exacerbating oxidative phosphorylation failure during energy-demanding states (25, 35).

## 4 Evidence of efficacy of exercise rehabilitation on HFpEF

The pathophysiological mechanism of HFpEF involves multisystem abnormalities, providing potential targets for exercise-based rehabilitation interventions. Although randomized controlled trials (RCTs) directly evaluating exercise rehabilitation remain limited, accumulating evidence indirectly supports its clinical utility. In the ejection fraction subgroup analysis of the REHAB-HF trial, while the prespecified interaction test lacked statistical significance (interaction *P* > 0.1), the intervention demonstrated a clinically meaningful improvement trend favoring the HFpEF subgroup. Specifically, Short Physical Performance Battery (SPPB) scores in rehabilitation-treated HFpEF patients increased by +1.9 points from baseline at 3-month follow-up, surpassing improvements observed in HFrEF counterparts, suggesting enhanced responsiveness to multidisciplinary rehabilitation strategies in this population (36).

The phenotypic heterogeneity of HFpEF necessitates individualized comprehensive care. As a cornerstone of lifestyle modification, exercise rehabilitation potentiates pharmacotherapy through synergistic blood pressure reduction and glycemic control (2). Observational cohort data indicate substantially elevated post-hospitalization venous thromboembolism (VTE) risk in HFpEF (adjusted HR = 3.13), with exercise potentially mitigating this risk via hemodynamic optimization and coagulation cascade modulation (31). Furthermore, 62% of new-onset atrial fibrillation (AF) cases exhibit high-risk HFpEF phenotypes (stratified by H2FPEF score), where structured exercise may attenuate arrhythmic progression through atrial unloading mechanisms (2, 37).

Future research must focus on three priorities: (1) developing phenotype-specific exercise prescriptions by stratifying subtypes (e.g., obesity or arterial stiffness-predominant phenotypes) for tailored regimens (37); (2) validating biomarker-guided efficacy, emphasizing exercise-enhanced cardiac function (e.g., global longitudinal strain) and refining risk stratification using tools like the H2FPEF score (2, 4, 8, 31); and (3) addressing adherence challenges in frail elderly, particularly in those with PH or ATTR-CM (19). While exercise may improve HFpEF prognosis via multimodal mechanisms, large RCTs are needed for confirmation. Integrating H2FPEF risk models with imaging biomarkers (e.g., speckle-tracking echocardiography) will advance precision rehabilitation strategies (2, 4, 5, 31).

## 5 Multimodal mechanisms of exercise rehabilitation in ameliorating HFpEF

Exercise rehabilitation improves the pathophysiological status of HFpEF patients through the synergistic effect of the central and peripheral multiple systems. Regarding central mechanisms, patients with HFpEF exhibit compromised cardiopulmonary reserve capacity and impaired ventriculoarterial coupling. Regular aerobic training may mitigate exercise-induced elevation in left ventricular filling pressure and abnormal pulmonary vascular pressures through reducing resting heart rate and ameliorating hemodynamic derangements (2). Exercise training enhances exercise-related cardiac output through coordinated optimization of preload regulation (e.g., reduced PCWP) and increased cardiac index (CI) (38). In high-risk heart failure patients, exercise-trained cohorts demonstrated significant reductions in PCWP during mild exercise (25 W), while exhibiting increased CI from 2.9 to 3.4 L/min/m² (39). These hemodynamic adaptations are mechanistically linked to enhanced cardiac reserve capacity, potentially involving improved cardiomyocyte calcium handling and optimized ventriculoarterial coupling (40, 41). Concurrently, exercise training restores endothelium-dependent vasodilation capacity (manifested as 2.5%–4.1% improvement in flow-mediated dilation) and ameliorates peripheral vascular resistance (41, 42), mechanisms associated with enhanced nitric oxide bioavailability, attenuation of oxidative stress markers (e.g., malondialdehyde), and improved endothelial progenitor cell functionality (43).

The peripheral mechanism in HFpEF is fundamentally characterized by skeletal muscle structural degeneration and metabolic remodeling. In HFpEF patients, skeletal muscles consistently demonstrate three cardinal pathological features: a 20%–50% reduction in capillary density, impaired mitochondrial oxidative phosphorylation capacity, and dysregulated autophagic flux (44, 45). This myopathic phenotype manifests clinically as mitochondrial dysfunction coupled with microcirculatory disturbances, collectively contributing to diminished exercise tolerance. Notably, exercise-based rehabilitation has been shown to ameliorate peripheral oxygen utilization through dual mechanisms: enhancing skeletal muscle oxidative metabolic capacity and stimulating angiogenesis (2). At the systemic level, exercise exerts metabolic-inflammatory regulatory effects on core risk factors including obesity and insulin resistance. Specifically, it reduces visceral adiposity deposition, suppresses proinflammatory cytokine release (e.g., IL-6 and TNF-α), and improves both endothelial function and insulin sensitivity through pleiotropic pathways (2, 5).

The multi-system synergistic interactions confer substantial clinical benefits in HFpEF management. Exercise rehabilitation induces a 35–50 m improvement in 6 min walking distance and 15–20-point elevation in KCCQ scores, demonstrating both functional and quality-of-life enhancements (46, 47). Beyond physiological adaptations, the therapeutic effects involve psychoneuroendocrine modulation, including anxiety alleviation through autonomic nervous system rebalancing (evidenced by increased heart rate variability) and reinforcement of self-efficacy (46). Crucially, longitudinal exercise interventions reduce cardiovascular hospitalization rates by 20%–30%, achieved via multi-organ protective mechanisms: suppression of systemic inflammation (0.5–1.2 mg/dl decrease in high-sensitivity C-reactive protein), enhancement of vascular compliance (8%–12% increase in carotid artery distensibility), and optimization of cardiopulmonary coupling efficiency (48) (Figure 1).

## 6 Optimization strategies for exercise rehabilitation in HFpEF

The optimization of exercise rehabilitation in HFpEF necessitates individualized, multidimensional intervention strategies, and prioritizes phenotype-driven precision therapeutics. Regarding exercise modality selection, combined endurance-resistance training should be tailored to phenotypic characteristics: endurance training (e.g., walking, cycling) significantly enhances peak oxygen uptake (VO2peak), while resistance training improves peripheral metabolic capacity via skeletal muscle functional augmentation—particularly critical for elderly patients with sarcopenia (49, 50). Meta-analytic evidence demonstrates that combined training improves both 6-minute walking distance and diastolic function parameters (e.g., E/e’ ratio reduction) (49). While older female phenotypes emphasize resistance and balance training (51, 52). In HFpEF subgroups with PH or respiratory muscle weakness, low-intensity inspiratory muscle training coupled with functional electrical stimulation (FES) safely optimizes hemodynamics and exercise tolerance. Notably, while high-intensity interval training (HIIT) exhibits proven efficacy in HFrEF, its application in HFpEF requires meticulous intensity titration based on baseline cardiopulmonary exercise testing (CPET) metrics (e.g., anaerobic threshold, VO2peak) (53).

Optimizing exercise prescription necessitates a delicate balance between safety and therapeutic efficacy. Current evidence supports moderate-intensity exercise regimens (40%–80% heart rate reserve) administered 3–5 sessions per week with 30–60 minutes per session, demonstrating that sustained implementation (>12 weeks) yields significant improvements in peak oxygen uptake (VO2peak) (mean increase: +2.72 ml/kg/min; 95% CI: 2.1–3.3) and enhanced quality-of-life metrics (e.g., KCCQ score *Δ*+8–12 points) (54). For obese HFpEF phenotypes, aerobic exercise combined with caloric restriction (e.g., ≥200 min/week moderate activity) demonstrates synergistic metabolic benefits (55).

For patients with HFpEF and AF, exercise should prioritize heart rate control (50%–70% max HR) to prevent ventricular rate escalation. Structured aerobic training (150 min/week) improves QoL (↑20%–30%) and LV function (LVEF ↑3%–5%) despite AF-related limitations (56). Elderly females require fall risk mitigation and anticoagulant safety evaluation (warfarin/DOACs) (56, 57).

Hypertension exacerbates HFpEF via LV hypertrophy and stiffness. Exercise rehabilitation requires integration with antihypertensives (ARNIs/SGLT2i), low-sodium diet, and monitored aerobic training (brisk walking/swimming) to ↓ peripheral vascular resistance (58–60). The REHAB-HF trial showed 6-minute walking distance gains (30–50 m) and frailty risk reduction with multi-domain rehabilitation (61, 62).

Intensity stratification proves critical: low-intensity training (40%–60% peak heart rate) is prioritized for patients with multiple comorbidities or severe PH, whereas moderate-high intensity (60%–80%) targets those with preserved functional reserves (CPET-derived anaerobic threshold >11 ml/kg/min) (63). Implementation safeguards include real-time heart rate monitoring via wearable technology and periodic 6-minute Walk Test and CPET to dynamically adjust workloads—strategies shown to reduce exertional adverse events by 38%–45% in vulnerable subgroups (64, 65). Meanwhile, echocardiography can serve as a follow-up assessment after exercise training, providing objective evidence for functional improvement and prognostic evaluation in HFpEF patients by assessing changes in LA pressure and pulmonary artery pressure (66). Emerging protocols further incorporate intervalized resistance training (2–3 sets, 60%–80% 1RM) to counteract sarcopenic progression while maintaining hemodynamic stability.

Multimodal interventions (e.g., exercise combined with SGLT2 inhibitors or nutritional protocols) may yield synergistic therapeutic effects (67, 68). SGLT2 inhibitors alleviate symptoms such as dyspnea and fatigue, enhance physical activity capacity and quality of life (QoL) scores, significantly reduce blood pressure, and lower the risk of heart failure hospitalizations and cardiovascular mortality (69–71). Multimodal intervention synergism emerges when combining exercise with SGLT2 inhibitors (e.g., dapagliflozin 10 mg/day) or omega-3 fatty acid supplementation (4 g/day EPA/DHA), showing additive improvements in ventricular compliance (E/e’ *Δ*−1.8) and systemic inflammation (hs-CRP *Δ*−0.6 mg/L) (52, 67, 72). Additionally, Glucagon-Like Peptide-1 (GLP-1) receptor agonists, such as liraglutide, not only promote weight loss but also improve cardiometabolic parameters and may confer benefits for patients with HFpEF (73).

Adherence management is critical for HFpEF rehabilitation efficacy. Multicomponent strategies (health education, goal-setting, biosensors) sustain ≥120 min/week exercise adherence while reducing anxiety (46). Home-based achieves outcomes comparable to center-based programs with 30%–45% cost reduction (72). Group CBT alleviates psychological burdens (depression *Δ*−2.4, *P* < 0.01) (45). Gamified mHealth platforms may enhance engagement via real-time feedback.

## 7 Challenges and future directions in exercise rehabilitation for HFpEF

Although exercise rehabilitation for heart failure with preserved ejection fraction (HFpEF) has demonstrated clinical benefits, it continues to face multiple challenges. First, unlike HFrEF, HFpEF lacks exercise-induced improvements in hard endpoints like mortality or cardiovascular hospitalization (41). Current research predominantly focuses on short-term outcomes (3–6 months), such as enhanced exercise tolerance and quality-of-life metrics (74), but lacks evidence for long-term prognostic benefits (75). Secondly, the physiological mechanisms underlying exercise benefits remain partially elucidated. While exercise augments peak oxygen uptake and 6-minute walk capacity, its mechanistic interplay with left ventricular diastolic function,skeletal muscle mitochondrial biogenesis, and peripheral vascular adaptation requires deeper interrogation (41, 76, 77). Furthermore, HFpEF patients are predominantly elderly, female, and often present with multiple comorbidities (e.g., obesity, diabetes mellitus, atrial fibrillation, hypertension), necessitating phenotype-driven, personalized, and multidimensional therapeutic approaches. Multimodal regimens integrating aerobic, resistance, and HIIT training with caloric restriction, SGLT2 inhibitors, and GLP-1 receptor agonists may yield superior therapeutic outcomes.

Routine CPET faces logistical challenges, including limited availability, cost, and patient compliance. While CPET may serve as an optional adjunct in specialized cardiac rehabilitation centers, alternative assessments such as the 6-minute walk test can be prioritized in resource-limited settings (78).

Infrastructure gaps persist: 78% of trials are hospital-based, and home/community models show lower adherence (58% vs. 85%) (77, 79). Unresolved debates on exercise modality (HIIT vs. MICT), frequency (3–5 vs. 5–7 sessions/week), and duration (30–60 vs. 20–45 min/session) contribute to guideline adherence <40% in real-world settings (80).

Future HFpEF research must achieve dual breakthroughs in mechanistic elucidation and technological innovation. Firstly, core exercise-mediated mechanisms, peripheral endothelial function, skeletal muscle mitochondrial metabolism, and oxygen utilization, require validation via multimodal imaging (STE, CMR T1 mapping) and biomarkers (NT-proBNP) (41, 76, 81). Secondly, personalized rehabilitation protocols require phenotypic stratification integrating clinical profiles (inflammatory/metabolic biomarkers) and energy metabolism gene expression (82–84), combined with wearable biosensors and tele-rehab platforms for real-time monitoring (77, 79).

Multimodal approaches, including high-intensity interval training (HIIT), resistance/flexibility training, and home-based models, are pivotal for HFpEF rehabilitation. HIIT enhances peak oxygen uptake (VO2peak) but requires hemodynamic safety validation (PCWP <25 mmHg) in elderly patients (85, 86). While HIIT combined with resistance training benefits HFrEF (2), HFpEF evidence remains limited, necessitating supervised trials with rigorous monitoring. Resistance/flexibility training combats sarcopenia (85). Home-based programs (e.g., REACH-HFpEF) improve accessibility but lack long-term efficacy data (87). Interdisciplinary integration—combining Mediterranean diets, cognitive therapy, and AI-driven “exercise-pharmacology-behavior” networks (e.g., REVERSE-HFpEF trial)—shifts management from symptom relief to disease modification (80, 84).

Bridging evidence gaps necessitates large-scale trials assessing exercise impacts on mortality and rehospitalization (41, 74). Inclusive enrollment of underrepresented groups (women, octogenarians, multimorbid patients) is critical (75, 83). Only through interdisciplinary collaboration, precision phenotyping, and technological innovation can we overcome the therapeutic challenges of HFpEF, ultimately improving patients’ functional status and long-term prognosis.

## 8 Conclusion

HFpEF, a multisystem disorder, demands personalized rehabilitation. Exercise improves functional capacity (VO2peak), quality of life, and hemodynamics via cardiac-skeletal adaptations and anti-inflammatory effects, yet lacks robust mortality/hospitalization reduction. Heterogeneous subphenotypes (obese, hypertensive, AF, frail, amyloidosis) require precision strategies integrating phenomapping (H2FPEF), biomarkers, and digital tools. In the future, large trials validating hard endpoints, home-based multimodal interventions, and AI-driven dynamic dosing to transition from symptom relief to disease modification.
